# Medicinal Earthworm: Speciation and Bioaccessibility of Arsenic and Its Potential Health Risks

**DOI:** 10.3389/fphar.2022.795530

**Published:** 2022-03-31

**Authors:** Yaolei Li, Hailiang Li, Ke Zan, Ying Wang, Tiantian Zuo, Hongyu Jin, Bing Zhang, Shuangcheng Ma

**Affiliations:** ^1^ School of Chinese Pharmacy, Beijing University of Chinese Medicine, Beijing, China; ^2^ National Institutes for Food and Drug Control, Beijing, China

**Keywords:** earthworm, arsenic species, inorganic arsenic, human health risk assessment, HPLC-ICP-MS

## Abstract

Arsenic in environmental health has caused public concerns all over the world. However, high levels of arsenic residues in medicinal animals have not received enough attention. Medicinal earthworms are consumed widely in China, but its arsenic potential health risks to humans are unknown. This work investigated the total concentration, bioaccessibility, and speciation of arsenic in earthworms by ICP-MS and HPLC-ICP-MS to evaluate its potential health risks to humans. Arsenic was found in all earthworms at concentrations ranging from 0.4 to 53.6 mg kg^−1^. The bioaccessibility of arsenic (bAs) varied significantly and ranged from 12.1 to 69.1%, with inorganic arsenic (iAs, including As(III) and As(V)) as the predominant species. Furthermore, a small amount of arsenobetaine (AB) was found. The estimated daily intake dose (EDI), hazard quotient (HQ), and carcinogenic risk (CR) of arsenic in most of the samples exceeded the safe threshold level. Results from this study indicated that the potential health risks by the consumption of earthworms may not be negligible. Herein, recommendations for the use of earthworms and regulatory recommendations for arsenic limit standards were proposed. This study reminds us that more control and monitoring of arsenic in medicinal animals should be carried out.

## Introduction

Arsenic in environmental health has caused public concerns in the world. Human exposure to arsenic is mainly through water and food (Yan et al., 2017; Ma et al., 2017). Among them, the traditional Chinese medicine (TCM) with a high arsenic content is the main way for human exposure to arsenic (Li et al., 2019; Liu et al., 2018). In recent years, higher arsenic residues in medicinal animals have attracted worldwide attention because these different species of arsenic are often absorbed into the food chain by animals and gradually passed to the end consumer, which leads to potential health risks (Wang Y et al., 2018). Most animal medicines that are in direct contact with the environment have arsenic residues, which leads to the accumulation of metal elements in their own tissues (Sánchez-Virosta et al., 2018; Peng et al., 2017). Earthworms are a typical animal medicine representative. The frequency of the use of medicinal earthworm is very high in China, and the arsenic’s potential health risks to humans are unknown. Until now, most of the limited regulations are for botanicals in the 2020 edition of the Chinese Pharmacopoeia (Chinese Pharmacopoeia, 2020), but only five medicinal animals such as leech are considered. Earthworms, being mainly distributed in Alpine regions in Shanghai and Guangzhou of China, have a long history of use in TCM. It is also an important ecological animal that plays an important role in the health of soil ecosystems by improving soil texture, enhancing decomposition, aeration, and water permeability (Geiszinger et al., 1998; Wang et al., 2019). The metal elements enriched in the earthworms serve as prey for the transfer of elements from soil throughout the food chain (Saxe et al., 2001; Ozaki et al., 2019). In-depth research is needed to assess potential health risks.

Most researchers focus on the correlation between arsenic and soil in earthworms in many studies. The scientists used the total arsenic (tAs) in the earthworm to reflect the arsenic contamination in soil, which was used as an indicator of soil pollution (Wang Z et al., 2019). The study found that when the concentration of arsenic in soil ranges from 16 to 348 mg kg^−1^, the arsenic content in the earthworms ranges from 6 to 239 mg kg^−1^ (Pass et al., 2014). Toxicological data indicate that adult earthworms exposed to arsenic up to 2,000 mg kg^−1^ (Langdon et al., 2009; Anderson et al., 2013) resulted in higher mortality. It was found that arsenic in earthworms was mainly present in the body cavity fluid (Porfido et al., 2019). In addition, many studies have focused on the relationship between arsenic and intestinal flora in the earthworm (Pass et al., 2014; Wang et al., 2019; Zhou et al., 2019).

Arsenic in medicinal earthworms has received little attention. Previous studies have focused on the bioaccumulation of metal elements in soil by earthworms, and only a few of these studies have detected tAs (Geiszinger et al., 1998; Moriarty et al., 2009). Some researchers have also analyzed five traces of organic arsenic such as AB, MAV, DMAV, TMAO, and arsenosugar 1 and arsenosugar 2 (Button et al., 2011). However, all these studies are not based on simulated *in vivo* digestion and do not reflect the real health risks of arsenic in medicinal earthworms. Arsenic in the earthworm must be dissolved in the gastrointestinal tract before it is absorbed into the blood, which may cause toxic effects (Ečimović et al., 2018). Hence, it is necessary to reveal the bioaccessible arsenic (bAs) species in the earthworm.

Arsenic toxicity was usually overestimated by considering the tAs concentration only in previous studies (Zhu et al., 2013). In fact, the species of arsenic is closely related to its toxicity (Wang et al., 2016; Xiao et al., 2018). Inorganic arsenic (iAs) is generally more toxic than organic arsenic (oAs) (Li et al., 2019). Hence, the hypothesis of this study is that arsenic and its speciation in medicinal earthworm need to be clarified, and bioaccessible arsenic needs to integrate the characteristics of earthworm use for health risk assessment. In this study, medicinal earthworms were investigated for the total concentration, bioaccessibility, and speciation of arsenic by inductively coupled plasma mass spectrometry (ICP-MS), high-performance liquid chromatography, and inductively coupled plasma mass spectrometry (HPLC-ICP-MS). The potential health risks to humans were evaluated according to the result. Meanwhile, recommendations for the use of earthworms and regulatory recommendations for arsenic limit standards were proposed. This study contributed to the determination of toxicity, safety evaluation, and risk assessment of arsenic in medicinal earthworms.

## Materials and Methods

### Standards and Reagents

Ultrapure water (18.2 MΩ cm, Direct-Q 3, Millipore, Bedford, United States) was used in the preparation of standards, mobile phase, and extraction solutions. Nitric acid (HNO_3_, 65.0%) was of ultrapure quality (Merck, Munchen, Germany). Ammonium carbonate ([NH_4_]_2_CO_3_, analytical reagent grade) and purified pepsin were obtained from Beijing Chemical Reagent Co. (Beijing, China). The other reagent chemicals used were of analytical reagent grade or better. Stock solutions of AB, methylarsonic acid (MMA), dimethylarsinic acid (DMA), arsenocholine (AsC), arsenite (As(III)), and arsenate (As(V)) were purchased from the National Institute of Metrology (Beijing, China). The tAs standard solution (100.0 μg ml^−1^) was obtained from the National Institutes for Food and Drug Control. Standard working solutions of AB, As(III), AsC, DMA, MMA, and As(V) were prepared by diluting stock solutions immediately before use. The germanium standard liquid (Agilent Technologies, Folsom, CA, United States) was used as an internal standard to ensure the stability of signals.

### Sample Collection and Preparation

A total of thirty earthworms were purchased from a pharmacy in 2018 in Beijing city or traditional herbal medicine markets (Table 1). These samples weighed approximately 50 to 100 g. All samples were authenticated by Mr. Shuai Kang, who was an associate researcher on the identification of medicinal materials in the National Institutes for Food and Drug Control (NIFDC). The sample was pulverized and passed through a sieve of 0.3 mm to obtain powder. All sample powders were stored at 4°C until analysis. The voucher specimens were deposited in the NIFDC, Beijing, China.

**TABLE 1:** Sample information.

Code	Location	Source	Code	Location	Source
1	Shanghai	Market (Bozhou)	16	Guangdong	Pharmacy (Beijing)
2	Hainan	Market (Bozhou)	17	Shanghai	Pharmacy (Beijing)
3	Shanghai	Market (Bozhou)	18	Jilin	Market (Bozhou)
4	Anhui	Market (Bozhou)	19	Anhui	Market (Bozhou)
5	Shanghai	Market (Bozhou)	20	Shanghai	Pharmacy (Beijing)
6	Shanghai	Market (Bozhou)	21	Shanghai	Market (Bozhou)
7	Guangdong	Market (Bozhou)	22	Guangdong	Market (Bozhou)
8	Guangxi	Market (Hehuachi)	23	Guangdong	Market (Hehuachi)
9	Shanghai	Market (Hehuachi)	24	Shanghai	Pharmacy (Beijing)
10	Guangxi	Market (Hehuachi)	25	Anhui	Market (Bozhou)
11	Shanghai	Market (Anguo)	26	Guangzhou	Pharmacy (Beijing)
12	Shanghai	Pharmacy (Beijing)	27	Guangdong	Pharmacy (Beijing)
13	Shanghai	Market (Bozhou)	28	Guangdong	Pharmacy (Beijing)
14	Guangxi	Pharmacy (Beijing)	29	Hebei	Market (Anguo)
15	Fujian	Market (Hehuachi)	30	Hebei	Market (Anguo)

### Bioaccessible Arsenic Extraction

Approximately 0.5 g of the sample powder was weighed into a 50-ml polyethylene centrifuge tube with addition of 10 ml simulated gastric juice. Briefly, the simulated gastric juice was prepared using 10 g of purified pepsin and 16.4 ml of diluted nitric acid diluted to 1,000 ml with deionized water. After sealing with a lid, the tube was placed on a vortex apparatus for 1 min to mix the extractant and sample powders thoroughly, and then the mixture was extracted by shaking in a water bath at 37°C for 6 h. After centrifuging at 6,000 rpm for 5 min, the supernatant was decanted into a 15-ml polyethylene centrifuge tube and stored at 4°C. Prior to analysis, the solutions were filtered through a 0.45-μm cellulose acetate membrane.

### Total Arsenic Determination by ICP-MS

Samples used to determine the tAs were digested using a MARS 5 microwave digestion system (CEM, United States). Approximately 0.5 g of the sample was weighed into a PTFE digestion tube, and then 8 ml of HNO_3_ was added in sequence. The microwave digestion program was as follows: heating for 3 min to 120°C and holding for 3 min, heating for 2 min to 150°C and holding for 3 min, and heating for 2 min to 200°C and holding for 12 min. After cooling, the solution was then transferred to a polyethylene flask and diluted with the deionized water to 50 ml. All samples were filtered through 0.45-μm membrane filters before determination by ICP-MS (Agilent 7700X, Agilent Technologies Co., United States). The operating conditions for ICP-MS are summarized in Table 2.

**TABLE 2:** Operating conditions of HPLC and ICP-MS.

ICP-MS parameter			
RF power	1,550 W		
Plasma gas flow	15.0 L min^−1^		
Carrier gas flow	1.05 L min^−1^		
Isotopes monitored	^75^As		
Quadrupole bias	−16.0 V		
Octopole bias	−18.0 V		
Dwell time for each isotope	0.1 s		
**HPLC conditions**			
Analytical column	Dionex IonPac^TM^ AS7 anion exchange column (250 mm × 4.6 mm, 10 μm)		
Mobile phase A	H_2_O		
Mobile phase B	100 mM (NH_4_)_2_CO_3_		
Injection volume	10 μl		
Flow rate	0.8 (ml min^−1^)		
Column temperature	Ambient temperature		
Gradient program	Time (min)	A%	B%
	0–3	90–50	10–50
	3–4	50–0	50–100
	4–11	0	100
	11–13	0–90	100–10
	13–17	90	10

### Arsenic Speciation Analysis by HPLC-ICP-MS

High-performance liquid chromatography (HPLC, Agilent 1,260, Agilent Technologies Co., United States) coupled with ICP-MS was used for the determination of arsenic species in the extracted solutions. This method was further optimized on the basis of the previous study (Zuo et al., 2018). The detailed operating parameters for HPLC-ICP-MS are listed in Table 2. The separation of six arsenic species including AB, As(III), DMA, AsC, MMA, and As(V) was performed in an anion exchange column run. The signal was monitored and collected in the time-resolved analysis (TRA) mode, and the polyatomic interference (e.g., ^40^Ar^35^Cl^+^ at m/z 75) was eliminated by a collision/reaction cell (Jia et al., 2018).

### Analysis, Quality Assurance, and Quality Control

For quality assurance, a certified reference material (CRM), citrus leaves (National Institute of Metrology, Beijing, China), was used during the tA measurement by ICP-MS. The tA concentration in citrus leaves was 1.0 ± 0.06 mg kg^−1^, which agrees well with the certified value (1.1 ± 0.2 mg kg^−1^). For the speciation analysis, the methods were validated by calculating several quality parameters. The analytical performances of HPLC-ICP-MS are shown in Table 3. The limits of detection (LOD) for AB, As(III), DMA, AsC, MMA, and As(V) were 10, 30, 30, 10, 10, and 10 pg, respectively, with a linear range from 10^−1^ to 500 ng ml^−1^. The spiked recoveries for different arsenic species were in the range from 92.69 to 105.7%, with relative standard deviations (RSDs) in the range from 0.89 to 2.4% (n = 6). Meanwhile, a procedural blank was analyzed in this study.

**TABLE 3:** Analytical performances of HPLC-ICP-MS.

Arsenical	Linear equation	Linear range (ng ml−1)	*R* ^2^	LODs (pg)	Recovery			
					Original value (ng ml^−1^)	Amount added (ng ml^−1^)	Average recovery (%)	RSDs (%)
AB	y = 0.9974x + 0.9020	10–500	0.9999	10	ND^a^	103.31	105.7	0.50
As(Ⅲ)	y = 1.0104x - 3.6351	10–500	1.0000	30	2.84	100.00	100.5	2.4
DMA	y = 1.0031x - 1.0833	10–500	0.9999	30	ND	99.26	102.9	0.93
AsC	y = 1.0108x - 3.7885	10–500	0.9995	10	ND	101.57	105.7	1.3
MMA	y = 0.9994x + 0.1978	10–500	0.9999	10	ND	100.13	100.2	0.89
As(Ⅴ)	y = 0.8560x + 50.4050	10–500	1.0000	10	ND	99.80	92.69	1.5

a Not detected.

### Health Risk Assessment

In order to assess the health risks of arsenic in earthworms, the following formulas were used (USEPA, 1989):

$$EDI=\frac{C\times IR}{BW},$$ (1)

where EDI (μg kg^−1^⋅day) is the estimated daily intake of arsenic, C (mg kg^−1^) is arsenic concentration, IR (g day^−1^) is the earthworm intake for an adult from the Chinese Pharmacopoeia 2020 (Chinese Pharmacopoeia Committee, 2020), and BW (kg) is the body weight of the consumer. The values of IR and BW for consumers are 10.0 g day^−1^ and 60.0 kg, respectively.

$$HQ=\frac{EDI}{RfD}\times \frac{EF\times ED\times 10}{AT},$$ (2)

where HQ is the hazard quotient of arsenic, RfD is the oral reference dose (μg kg^−1^ day^−1^) with value 0.3 μg kg^−1^ day^−1^ (USEPA, 2015), EF is the exposure frequency (90 days year^−1^) (Zuo et al., 2019), ED is the exposure duration (20 y), AT is the average exposure time (25,550 days) (Liu et al., 2013; Liu et al., 2018), and 10 is a safety factor, which means that the amount of arsenic consumed by TCM and its preparations per day is not greater than 10% of the total daily exposure (including food and water). If the HQ＞1, toxic risk exists, with an increasing possibility as the value increases.

The carcinogenic health risk (CR) to iAs from earthworm consumption was evaluated from the following equation:

$$CR=EDI\times SF\times \frac{EF\times ED\times 10}{AT},$$ (3)

where SF (kg day μg^−1^) is the cancer slope factor set by the USEPA only for iAs (USEPA, 2015; Jia et al., 2018; Liu et al., 2018). The SF value for iAs was 1.5 × 10^−3^ kg day μg^−1^.

## Results and Discussion

### Optimization of Chromatographic Separation Conditions

The HPLC-ICP-MS method in this study was further optimized. First, anion exchange columns were selected with mobile phases ([NH_4_]_2_CO_3_ and water). We compared the two columns (Dionex IonPac^TM^ AS7 and PRP-X100) and found that only five arsenicals were separated with the PRP-X100 column. All these arsenicals have broad chromatographic peaks and some even trailing. Conversely, the Dionex IonPac^TM^ AS7 column had strong elution ability, and six arsenicals were separated well.

Second, the salt of the mobile phase was screened. We investigated the effects of the same concentration of (NH_4_)_2_CO_3_, NH_4_H_2_PO_4_, (NH_4_)_2_HPO_4_, and CH_3_COONH_4_ solutions on the separation. We found that (NH_4_)_2_CO_3_ has a good effect on the separation of six arsenicals, and the peak shape well and the baseline noise lower. Furthermore, the ratio of (NH_4_)_2_CO_3_ was explored using three levels (50, 100, and 150 mM L^−1^). The results show that change in the salt ratio has a greater impact on the retention time of DMA and the least on AB. The level 100 mM L^−1^ was selected according to the optimal conditions.

Finally, different flow rates were optimized. The experiments were carried out in flow rates of 0.5, 0.8, 1, and 1.2 ml min^−1^, respectively. Studies have shown that the baseline noise became larger as the flow rate increased; however, the As(III) peak tailed when the flow rate decreased. In sum, the separation and elution effect of 0.8 ml min^−1^ was better.

### Analysis of Total Arsenic and Arsenic Species in Earthworms

**FIGURE 1:**
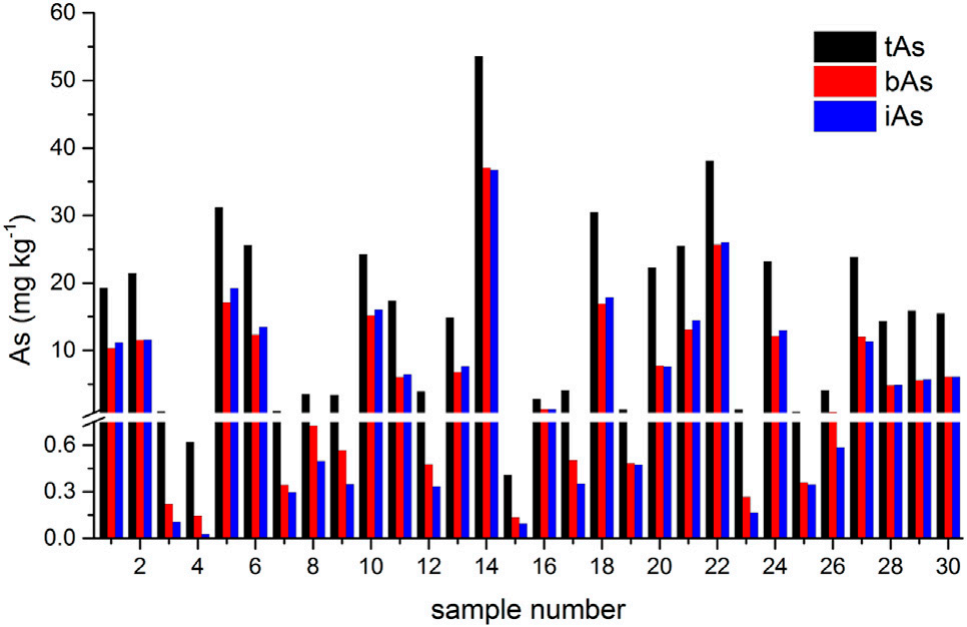
Results of total arsenic (tAs), bioaccessible arsenic (bAs), and inorganic arsenic (iAs) analyzed in earthworms.

**FIGURE 2:**
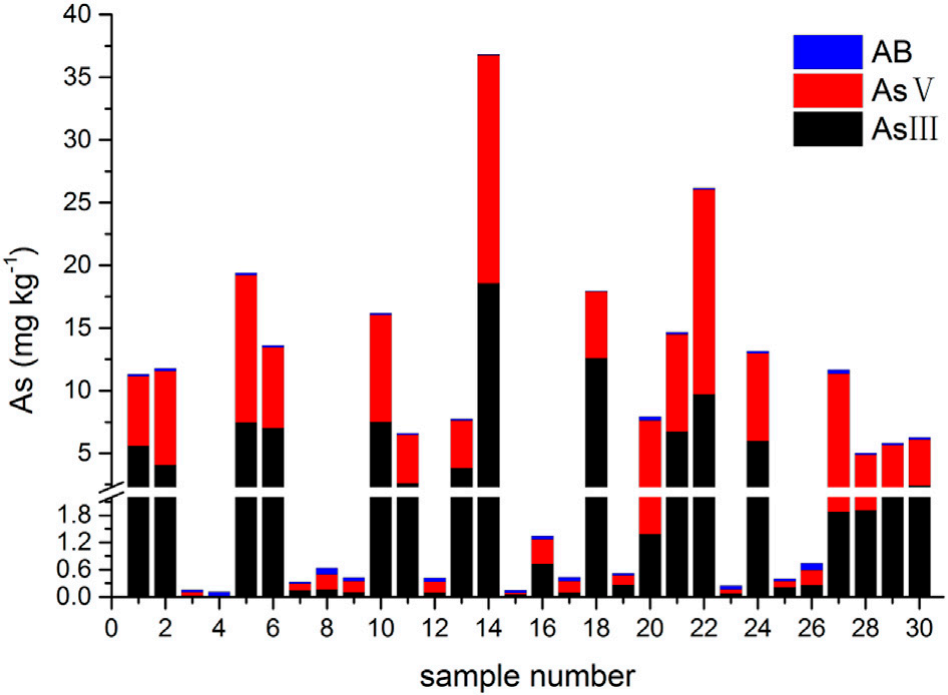
Arsenic species (arsenobetaine (AB) and arsenite (As(III)) and arsenate (As(V)) from bioaccessibility in earthworms.

**FIGURE 3:**
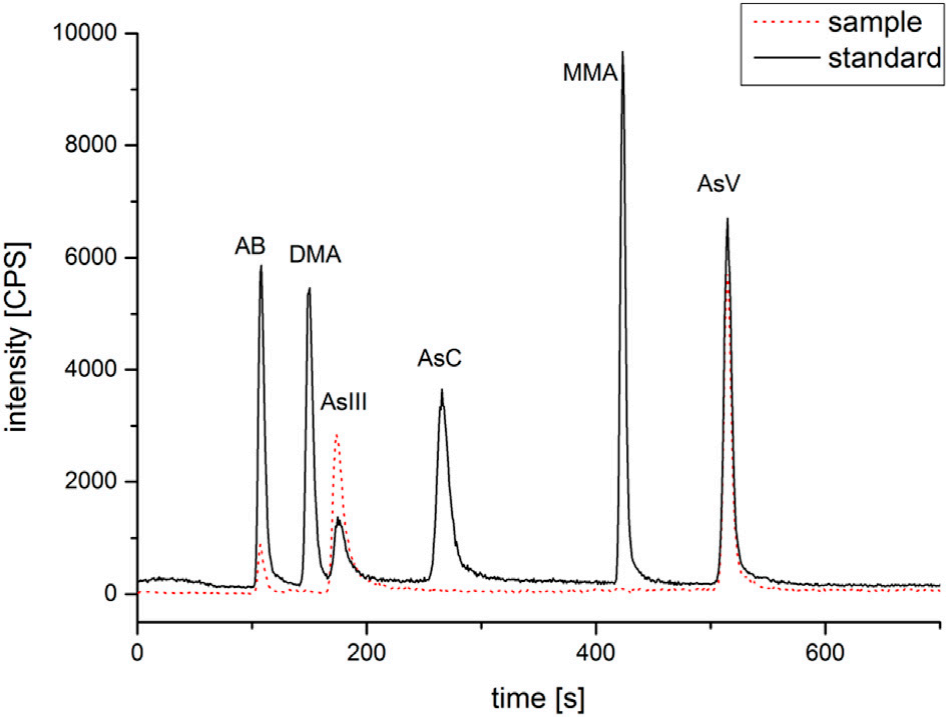
Representative chromatogram of arsenic speciations (arsenobetaine (AB), methylarsonic acid (MMA), dimethylarsinic acid (DMA), arsenocholine (AsC), and arsenite (As(III)) and arsenate (As(V)) with sample (No. 14) and standard (100 ng ml^−1^).

**FIGURE 4:**
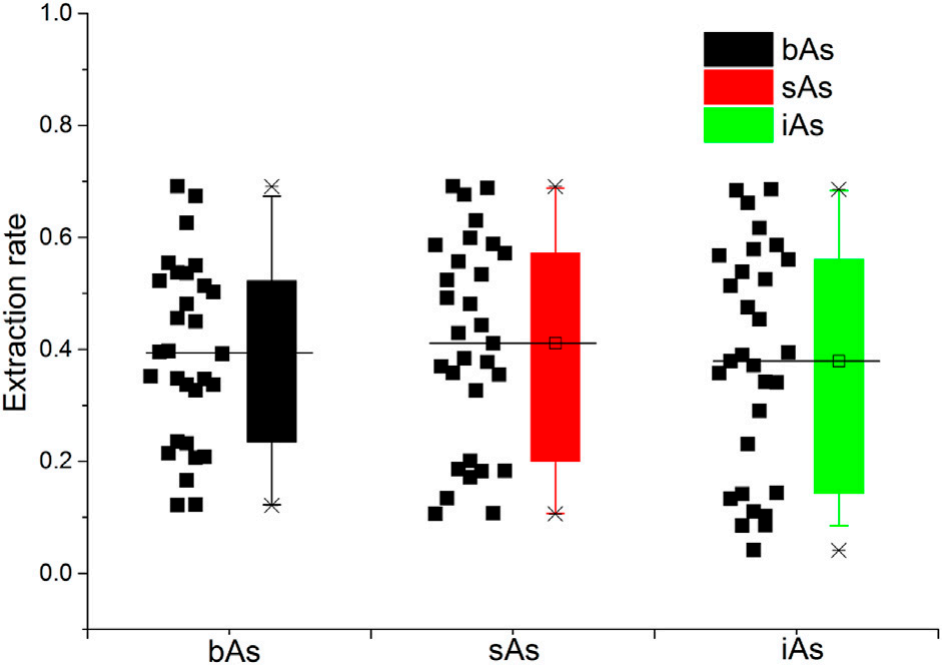
Extraction rates using simulated gastric juice of bioaccessible arsenic (bAs), speciation of arsenic (sAs), and inorganic arsenic (iAs).

The results of tAs, bAs, and iAs analyzed in earthworms are shown in Figure 1. The tA concentrations of a total of 30 earthworm samples range between 0.4 and 53.6 mg kg^−1^. Similarly, the range of bA concentrations are ranged from 0.1 to 37.1 mg kg^−1^ to iAs from 0.03 to 36.8 mg kg^−1^. Furthermore, the proportion of bAs to iAs is high in earthworms. iAs and AB were the only species detected in earthworms. As(III) and As(V) from bioaccessibility were the predominant species, with a small amount of AB (Figure 2). Also, the range of the As(III) concentration is from 0.02 to 18.6 mg kg^−1^ and that of As(V) from 0.01 to 18.2 mg kg^−1^. The concentration of AB is only between 0.04 and 0.3 mg kg^−1^, and other organic arsenic is not detected. A representative chromatogram is shown in Figure 3. The analysis of bAs, speciation of arsenic (sAs) and iAs, and the extraction rates using simulated gastric juice were 39, 41, and 38%, respectively (Figure 4). It should be noted that almost all bAs are iAs. This result suggests that health risks of arsenic in earthworms must be assessed.

**FIGURE 5:**
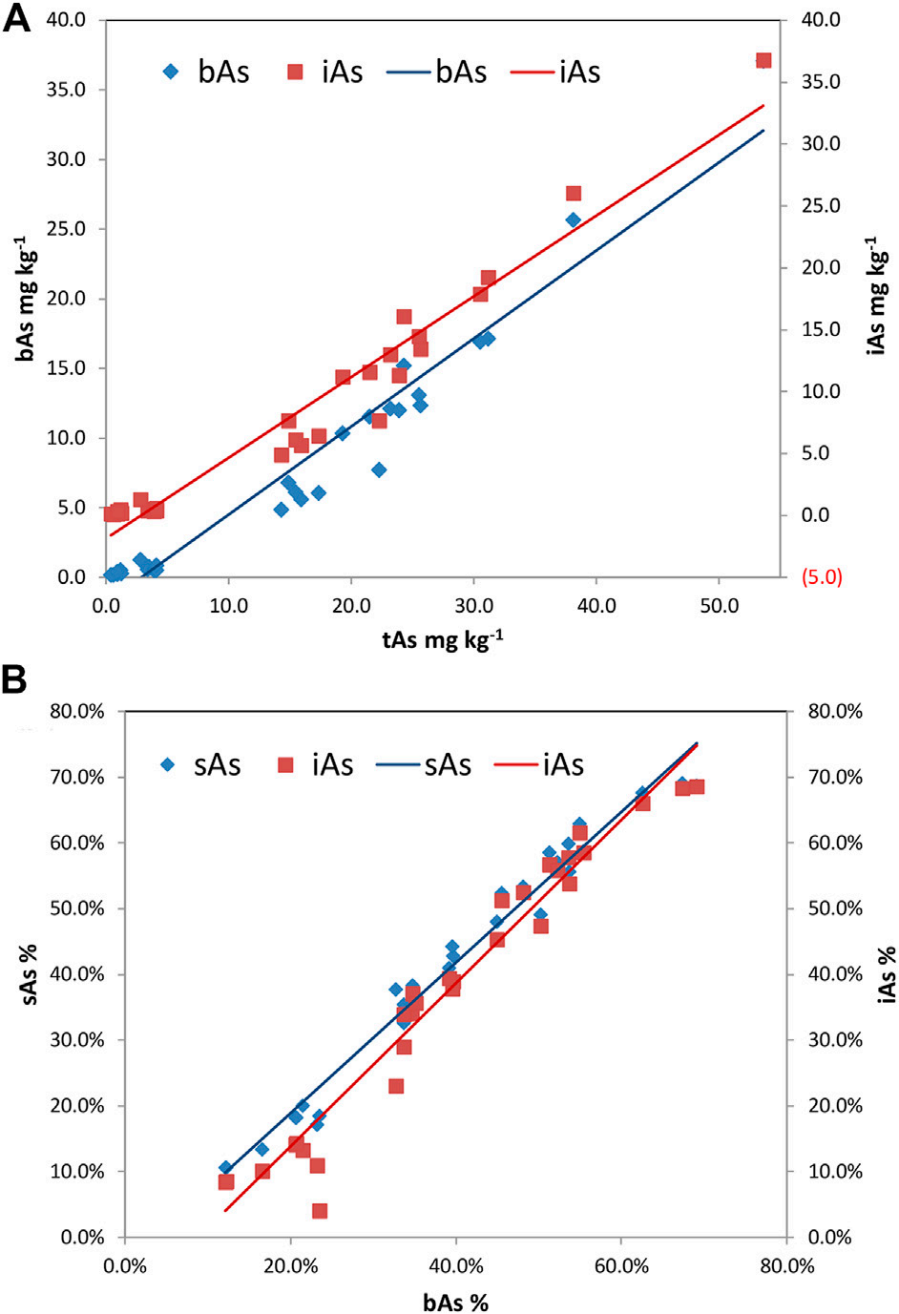
Correlations among total arsenic (tAs), bioaccessible arsenic (bAs), and inorganic arsenic (iAs) in earthworm [**(A)** bAs: y = 0.6323x-1.8247, r = 0.974, *p*＜0.01; **(A)** iAs: y = 0.6522x-1.8705, r = 0.977, *p*＜0.01; **(B)** sAs: y = 1.1457x-0.0404, r = 0.976, *p*＜0.01; and **(B)** iAs: y = 1.2417x-0.1102, r = 0.988, *p*＜0.01].

The relationships of tAs–bAs and tAs–iAs were determined by Pearson’s correlation analysis. Positive correlations with the significance at 0.01 levels were determined between the concentrations of tAs and most arsenic species. A positive correlation was found between bAs and tAs (r = 0.974, *p* < 0.01), as shown in Figure 5A, indicating the concentration of bAs was proportional to that of tAs. Similarly, iAs and tAs showed the same trend (r = 0.977, *p* < 0.01). Apart from the concentration, the percentages of sAs and iAs were used to investigate the correlation with bAs (Figure 5B). Again, positive correlations between the percentage of sAs and bAs (r = 0.976, *p* < 0.01) and iAs and bAs (r = 0.988, *p* < 0.01) indicated that the concentrations of sAs and iAs increased significantly with increasing bA percentage in earthworm samples.

In addition, arsenic may have different binding states due to the complexity of the animal drug matrix. For instance, arsenic was combined with proteins and polysaccharides, and the like. It is difficult for these biomacromolecules to pass through the ion exchange chromatography column due to the large molecular weight, so that the arsenic-bound compounds cannot be detected (Li et al., 2019). Hence, column recovery is also an important factor in the detection of arsenic species. Column recovery can be obtained by the ratio of arsenic detected to tAs extracted. In this study, the column recovery ranged from 80.8 to 115.4%. The recoveries ranged from 70 to 120%, indicating that bAs was completely detected without other forms of arsenic-bound compounds.

### Binding State of Arsenic and Accumulation in Earthworms

In this study, the bioaccessible arsenic of earthworms accounts for an average of 39%, and almost all of them were iAs. In general, trivalent arsenic is easily associated with biothiols, including mammalian metallothionein (MT), a ubiquitous sulfur-rich MT that coordinates a variety of metals (Ngu and Stillman, 2006). Until now, it has been found that the coordination of arsenic in biological tissues is mainly As(III)-S, including non-high accumulation plants, invertebrates, human blood cells, and protozoa. These compounds may be involved in the accumulation and transport of arsenic in organisms (Moriarty et al., 2009). Some evidence indicated that arsenic can induce the expression of MT in earthworms, which in turn can sequester arsenic in specific cells and tissues (Wang et al., 2016). In fact, the chelation of metals is promoted by chlorogenic cells. Chlorogenic cells account for 30% of cells in the body cavity, and the concentration of MT in the body cavity fluid is proportional to the overall MT content (Allegretta et al., 2017). In sum, we speculated that the prototype of arsenic in earthworms is combined with MT.

Due to the metabolic needs and the enrichment ability of various elements, earthworms have lived long in the soil, and harmful elements such as arsenic are absorbed along with their growth, which may cause arsenic enrichment and accumulation (Buch et al., 2017). However, according to literature reports, there is no direct correlation between iAs in earthworms and soil (Geiszinger et al., 1998; Moriarty et al., 2009). It is speculated that the demethylation of oAs in the absorption of arsenic by earthworms may lead to highly toxic iAs. Earthworms mainly absorb and enrich arsenic through the skin and intestines (Button et al., 2012). It has been found that the sources of oAs observed in earthworms include the biotransformation of iAs in the soil by earthworms (Langdon et al., 2001). Intestinal microbes are accumulated from the gut intestines after transformation into As(V) (Langdon et al., 2012).

AB may be derived from the biotransformation of arsenic, the secondary metabolite of earthworms (Langdon et al., 2003), which presumably converts highly toxic iAs into less-toxic oAs in earthworms (Wang et al., 2016). Earthworms reduce the bioavailability of arsenic by adsorbing and combining As(V). Meanwhile, the effect of arsenic on the intestinal flora is alleviated, thereby preventing the accumulation of iAs and tAs in the intestinal tract (Wang et al., 2019). This combination of other substances indirectly reduces the toxicity of arsenic and increases the tolerance of earthworms. The arsenic-bound states are unstable under acidic or alkaline conditions. Almost all of them are easily released as free iAs in the human body environment and have many potential health risks.

### Health Risk Assessment

**FIGURE 6:**
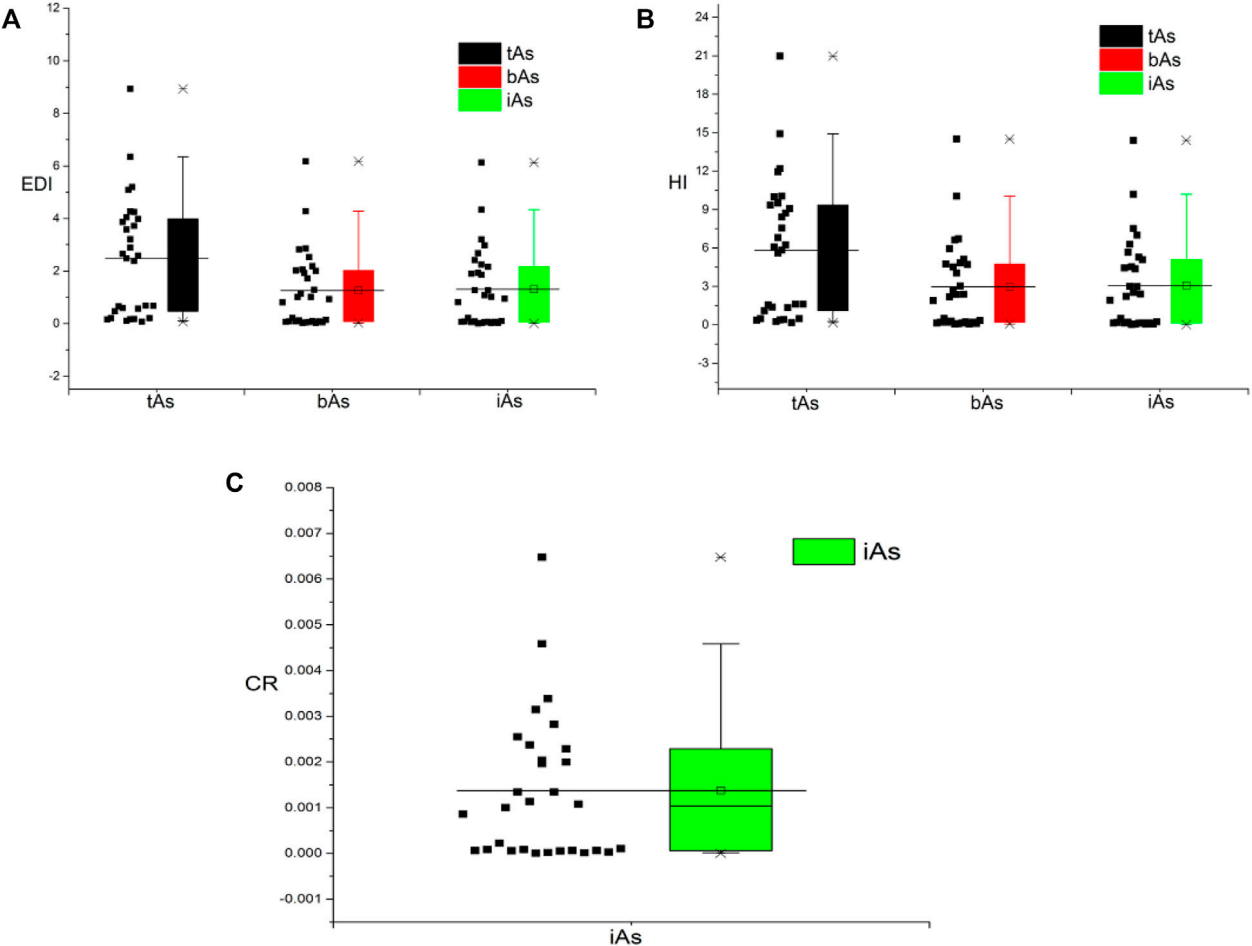
Total arsenic (tAs), bioaccessible arsenic (bAs), and inorganic arsenic (iAs) of health risks assessment with estimated daily intake dose (EDI, **A**), hazard quotient (HQ, **B**), and carcinogenic risk (CR, **C**).

The potential chronic health risks associated with long-term exposure to arsenic from medicinal earthworm consumption were evaluated through the calculation of EDI, HQ, and CR. As shown in Figure 6A, the EDIs of the tAs, bioaccessible arsenic, and iAs were in the range from 0.1 to 8.9, 0.02 to 6.2, and 0.004 to 6.1 μg kg^−1^ d^−1^, with an average value of 2.5, 1.3, and 1.3, respectively. Among them, the percentages of earthworm’s EDI in the three groups exceeded the daily intake reference dose suggested by the USEPA (0.3 μg kg^−1^ d^−1^), which were 76.7, 56.7 and 56.7%, respectively. Therefore, the high EDI values of arsenic showed that the earthworms posed a threat to human health.

As listed in Figure 6B, the HQs for tAs for 30 earthworms were in the range from 0.2 to 21.0, and 23 of them were more than one, indicating potential health risk to the consumers (USEPA, 1989). HQs of both the bAs and iAs were in the range from 0.1 to 14.5 and 0.01 to 14.4, with those in 17 samples more than one.

According to Figure 6C, the CR value of iAs was between 4.5 × 10^−6^ and 0.0065, with an average value of 0.0014, and the CR value exceeded the specified range from 1 × 10^−6^ to 1 × 10^−4^ (Ma et al., 2016). This result represented that continuous exposure to iA contamination from consumption of medicinal earthworms would pose a potential carcinogenic risk to the patients.

### Recommendations for Use of Earthworms and Their Arsenic Management

Medicinal earthworms are consumed widely in China due to their specific pharmacological effect. It may be taken regularly by some patient groups. According to the source of arsenic in earthworms, it is difficult to improve and regulate the mechanism of arsenic accumulation. For its arsenic health risks in earthworms, it is related to the amount, time, and frequency of exposure. Consumers can only change the frequency or amount of use to avoid ingesting too much arsenic through the earthworms. Herein, we evaluated different health risks (HQs and CRs) using different frequencies and amounts of use for medicinal earthworms (Table 4). The results indicated that usage with 3.5 g day^−1^ or frequency with 1 month year^−1^ has no health risks for consumers (based on bA concentration and ED with 20 y). In this scenario, although there are carcinogenic health risks, it is significantly lower than before. Meanwhile, medicinal earthworms play a role in treating disease, and patients need to accept the health risks caused by side effects. It is different from food, and it is to treat diseases. Hence, usage with 3.5 g day^−1^ or frequency with 1 month year^−1^ can be used as a reference for clinicians’ and consumers’ medication guidance.

**TABLE 4:** Hazard quotient (HQ) and carcinogenic risk (CR) with different frequencies and amount of use for medicinal earthworms.

Usage/frequency	HQ	CR
1 g day^−1^	0.3	1 × 10^−4^
3.5 g day^−1^	1	5 × 10^−4^
10 g day^−1^	3	1 × 10^−3^
10 days year^−1^	0.3	1 × 10^−4^
1 month year^−1^	1	4 × 10^−4^
3 months year^−1^	3	1 × 10^−3^

The pollution of heavy metals in TCM is largely due to environmental pollution. It cannot be improved in a short time. Therefore, the development of a limited standard does not fundamentally solve the problem of arsenic pollution in medicinal earthworms. Arsenicals have a long half-life in the human body and tend to accumulate in the body during long-term exposure. Based on the earlier results from this study, we can modify the usage and dosage in the instruction manual and pharmacopeia standard. For instance, information such as the dose and frequency of use must be clear. In addition, the reasons for arsenic pollution in medicinal earthworms are further needed to be determined, and the whole process of medicinal earthworms production, including planting, irrigation, harvesting, and processing must be monitored.

Arsenic in medicinal earthworms is also closely related to clinics. It is difficult to reflect the true toxicity status under the complex system of TCM that only pays attention to the toxic ingredients and ignores the clinical factors. The clinical use of earthworms is complicated, and different drug compatibility, medication methods, medication cycles, and formulations are all related to the safety of arsenic in medicinal earthworms. First of all, there is a difference between earthworms alone and their compatibility. Second, the toxic components of earthworms are dissolved out during the decoction process. Third, taking the earthworm course of treatment has an effect on the toxic ingredients. Fourth, the different dosage forms of earthworms can absorb the toxic ingredients in the body. In addition to the aforementioned clinical factors, there are many factors that affect the effects of toxic ingredients, such as syndromes, age, and gender. In future research, we need to focus on the safety of arsenic in earthworms based on the effective treatment window and toxicity safety window of arsenic, combined with the method of use and the target to be treated.

## Conclusion

**FIGURE 7:**
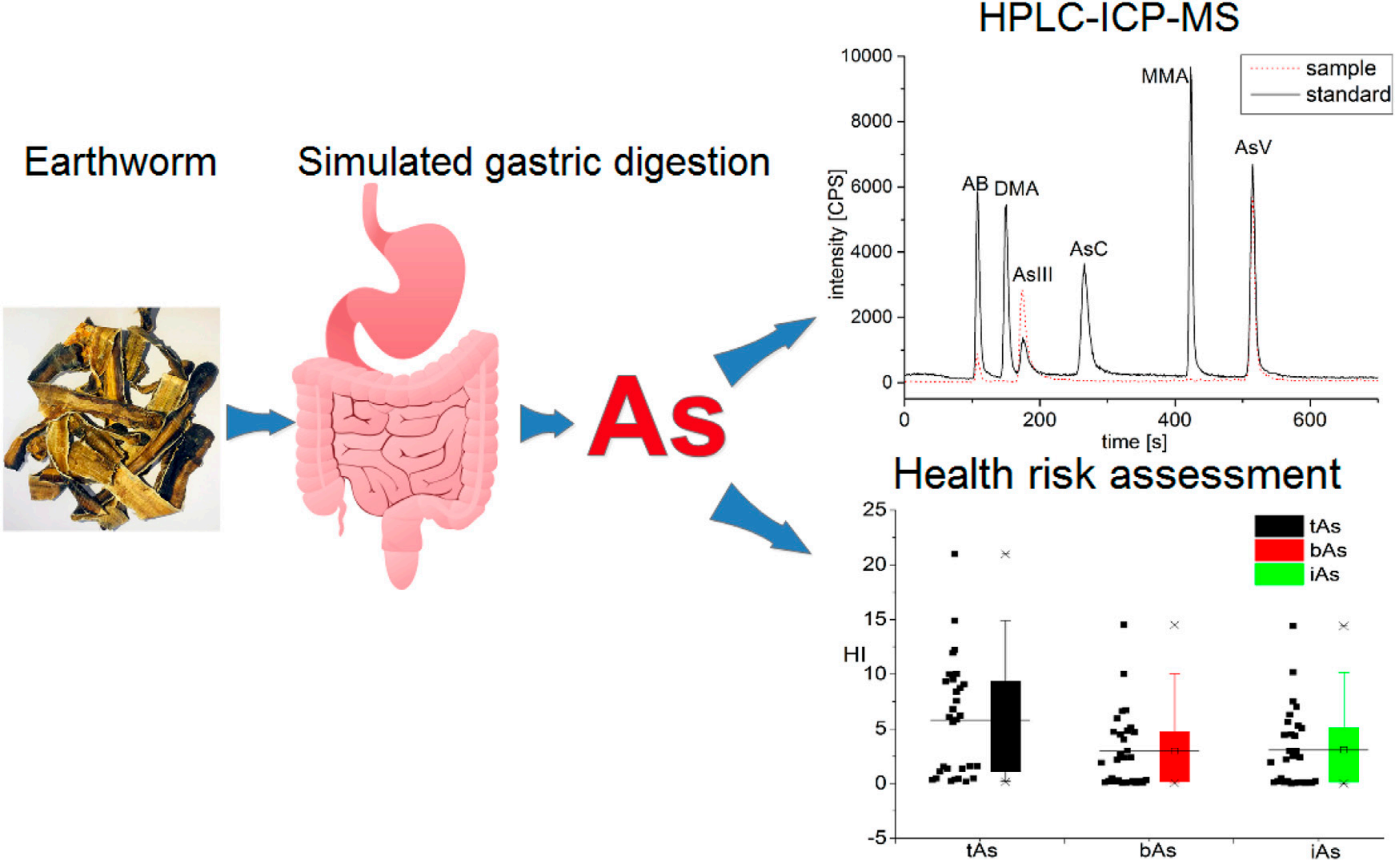
Speciation and bioaccessibility of arsenic and its potential health risks in medicinal earthworms.

Arsenic in TCM usually has caused public concerns in the world. In this study, we described the species of bioaccessible arsenic in medicinal earthworms (Figure 7). iAs were the predominant species in earthworms, with a small amount of AB. Furthermore, the potential health risk caused by the consumption of earthworms may not be negligible. In addition, many recommendations for the use of earthworms and regulatory were proposed. The toxicity, safety, and health risks of arsenic in medicinal earthworms were further clarified in this study. In the future, we will focus on arsenic pollution prevention and arsenic removal in medicinal earthworms to ensure its quality and safety.

## Data Availability

The original contributions presented in the study are included in the article/Supplementary Material, further inquiries can be directed to the corresponding authors.

## Abbreviations

As, arsenic; As(III), arsenite; As(V), arsenate; DMA, dimethylarsinic acid; MMA, methylarsonic acid; AsB, arsenobetaine; AsC, arsenocholine; sAs, speciation of arsenic; oAs, organic arsenic; iAs, inorganic arsenic; bAs, bioaccessible arsenic; tAs, total arsenic; EDI, estimated daily intake; HQ, hazard quotient; CR, carcinogenic risk, TCM, traditional Chinese medicine; HPLC-ICP-MS, high-performance liquid chromatography inductively coupled plasma mass spectrometry.

## References

[B1] Allegretta I. Porfido C. Panzarino O. Fontanella M. C. Beone G. M. Spagnuolo M. (2017). Determination of as Concentration in Earthworm Coelomic Fluid Extracts by Total-Reflection X-ray Fluorescence Spectrometry. Spectrochimica Acta B: At. Spectrosc. 130, 21–25. 10.1016/j.sab.2017.02.003

[B2] Anderson C. J. Kille P. Lawlor A. J. Spurgeon D. J. (2013). Life-history Effects of Arsenic Toxicity in Clades of the Earthworm Lumbricus Rubellus. Environ. Pollut. 172, 200–207. 10.1016/j.envpol.2012.09.005 23063995

[B3] Buch A. C. Brown G. G. Correia M. E. F. Lourençato L. F. Silva-Filho E. V. (2017). Ecotoxicology of Mercury in Tropical forest Soils: Impact on Earthworms. Sci. Total Environ. 589, 222–231. 10.1016/j.scitotenv.2017.02.150 28258750

[B4] Button M. Koch I. Reimer K. J. (2012). Arsenic Resistance and Cycling in Earthworms Residing at a Former Gold Mine in Canada. Environ. Pollut. 169, 74–80. 10.1016/j.envpol.2012.04.031 22683483

[B5] Button M. Moriarty M. M. Watts M. J. Zhang J. Koch I. Reimer K. J. (2011). Arsenic Speciation in Field-Collected and Laboratory-Exposed Earthworms Lumbricus Terrestris. Chemosphere 85 (8), 1277–1283. 10.1016/j.chemosphere.2011.07.026 21868054

[B6] Chinese Pharmacopoeia Committee (2020). Chinese Pharmacopoeia. Beijing, China: People's Press.

[B7] Ečimović S. Velki M. Vuković R. Čamagajevac I. Š. Petek A. Bošnjaković R. (2018). Acute Toxicity of Selenate and Selenite and Their Impacts on Oxidative Status, Efflux Pump Activity, Cellular and Genetic Parameters in Earthworm *Eisenia andrei* . Chemosphere 212, 890–897. 10.1016/j.chemosphere.2018.08 30145422

[B8] Geiszinger A. Goessler W. Kuehnelt D. Francesconi K. Kosmus W. (1998). Determination of Arsenic Compounds in Earthworms. Environ. Sci. Technol. 32 (15), 2238–2243. 10.1021/es980018y

[B9] Jia Y. Wang L. Ma L. Yang Z. (2018). Speciation Analysis of Six Arsenic Species in Marketed Shellfish: Extraction Optimization and Health Risk Assessment. Food Chem. 244, 311–316. 10.1016/j.foodchem.2017.10.064 29120787

[B10] Langdon C. J. Meharg A. A. Feldmann J. Balgar T. Charnock J. Farquhar M. (2012). Arsenic-speciation in Arsenate-Resistant and Non-resistant Populations of the Earthworm, Lumbricus Rubellus. J. Environ. Monit. 4, 603–608. 10.1039/B201366P 12196009

[B11] Langdon C. J. Morgan A. J. Charnock J. M. Semple K. T. Lowe C. N. (2009). As-resistance in Laboratory-Reared F1, F2 and F3 Generation Offspring of the Earthworm Lumbricus Rubellus Inhabiting an As-Contaminated Mine Soil. Environ. Pollut. 157, 3114–3119. 10.1016/j.envpol.2009.05.027 19501438

[B12] Langdon C. J. Piearce T. G. Feldmann J. Semple K. T. Meharg A. A. (2003). Arsenic Speciation in the Earthworms Lumbricus Rubellus and Dendrodrilus Rubidus. Environ. Toxicol. Chem. 22, 1302–1308. 10.1897/1551-5028(2003)022<1302:asitel>2.0.co;2 12785588

[B13] Langdon C. J. Piearce T. G. Meharg A. A. Semple K. T. (2001). Survival and Behaviour of the Earthworms Lumbricus Rubellus and Dendrodrilus Rubidus from Arsenate-Contaminated and Non-contaminated Sites. Soil Biol. Biochem. 33, 1239–1244. 10.1016/S0038-0717(01)00029-3

[B14] Li Y. Liu Y. Han X. Jin H. Ma S. (2019). Arsenic Species in *Cordyceps Sinensis* and its Potential Health Risks. Front. Pharmacol. 10, 1471. 10.3389/fphar.2019.01471 31866869 PMC6910106

[B15] Liu L. Zhang Y. Yun Z. He B. Zhang Q. Hu L. (2018). Speciation and Bioaccessibility of Arsenic in Traditional Chinese Medicines and Assessment of its Potential Health Risk. Sci. Total Environ. 619-620, 1088–1097. 10.1016/j.scitotenv.2017.11.113 29734587

[B16] Liu X. J. Zhao Q. L. Sun G. X. Williams P. Lu X. J. Cai J. Z. (2013). Arsenic Speciation in Chinese Herbal Medicines and Human Health Implication for Inorganic Arsenic. Environ. Pollut. 172, 149–154. 10.1016/j.envpol.2012.09.009 23063615

[B17] Ma J. Mi Y. Li Q. Chen L. Du L. He L. (2016). Reduction, Methylation, and Translocation of Arsenic in Panax Notoginseng Grown under Field Conditions in Arsenic-Contaminated Soils. Sci. Total Environ. 550, 893–899. 10.1016/j.scitotenv.2016.01.188 26851761

[B18] Ma L. Wang L. Tang J. Yang Z. (2017). Arsenic Speciation and Heavy Metal Distribution in Polished rice Grown in Guangdong Province, Southern China. Food Chem. 233, 110–116. 10.1016/j.foodchem.2017.04.097 28530555

[B38] Moriarty M. M. Koch I. Gordon R. A. Reimer K. J. (2009). Arsenic Speciation of Terrestrial Invertebrates. Environ. Sci. Technol. 43 (13), 4818–4823. 10.1021/es900086r 19673270

[B19] Ngu T. T. Stillman M. J. (2006). Arsenic Binding to Human Metallothionein. J. Am. Chem. Soc. 128 (38), 12473–12483. 10.1021/ja062914c 16984198

[B20] Ozaki S. Fritsch C. Valot B. Mora F. Cornier T. Scheifler R. (2019). How Do Richness and Composition of Diet Shape Trace Metal Exposure in a Free-Living Generalist Rodent, *Apodemus sylvaticus* . Environ. Sci. Technol. 53, 5977–5986. 10.1021/acs.est.8b07194 31002242

[B21] Pass D. A. Morgan A. J. Read D. S. Field D. Weightman A. J. Kille P. (2014). The Effect of Anthropogenic Arsenic Contamination on the Earthworm Microbiome. Environ. Microbiol. 17 (6), 1884–1896. 10.1111/1462-2920.12712 25404571

[B22] Peng H. Hu B. Liu Q. Li J. Li X. F. Zhang H. (2017). Methylated Phenylarsenical Metabolites Discovered in Chicken Liver. Angew. Chem. Int. Ed. Engl. 56 (24), 6773–6777. 10.1002/anie.201700736 28470989 PMC5573966

[B23] Porfido C. Allegretta I. Panzarino O. Laforce B. Vekemans B. Vincze L. (2019). Correlations between as in Earthworms' Coelomic Fluid and as Bioavailability in Highly Polluted Soils as Revealed by Combined Laboratory X-ray Techniques. Environ. Sci. Technol. 53, 10961–10968. 10.1021/acs.est.9b02310 31373803

[B24] Sánchez-Virosta P. Espín S. Ruiz S. Salminen J.-P. García-Fernández A. J. Eeva T. (2018). Experimental Manipulation of Dietary Arsenic Levels in Great Tit Nestlings: Accumulation Pattern and Effects on Growth, Survival and Plasma Biochemistry. Environ. Pollut. 233, 764–773. 10.1016/j.envpol.2017.10.113 29127934

[B25] Saxe J. K. Impellitteri C. A. Peijnenburg W. J. Allen H. E. (2001). Novel Model Describing Trace Metal Concentrations in the Earthworm, *Eisenia andrei* . Environ. Sci. Technol. 35 (22), 4522–4529. 10.1021/es0109038 11757611

[B26] USEPA (2015). Regional Screening Level (RSL) Summary Table.

[B27] USEPA (1989). Risk Assessment Guidance for Superfund: Volume I-Human Health Evaluation Manual (Part A).

[B28] Wang H.-T. Zhu D. Li G. Zheng F. Ding J. O’Connor P. J. (2019). Effects of Arsenic on Gut Microbiota and its Biotransformation Genes in Earthworm Metaphire Sieboldi. Environ. Sci. Technol. 53, 3841–3849. 10.1021/acs.est.8b06695 30875464

[B29] Wang Y Y. Wu Y. Cavanagh J. Yiming A. Wang X. Gao W. (2018). Toxicity of Arsenite to Earthworms and Subsequent Effects on Soil Properties. Soil Biol. Biochem. 117, 36–47. 10.1016/j.soilbio.2017.11.007

[B30] Wang Z. Cui Z. Liu L. Ma Q. Xu X. (2016). Toxicological and Biochemical Responses of the Earthworm *Eisenia fetida* Exposed to Contaminated Soil: Effects of Arsenic Species. Chemosphere 154, 161–170. 10.1016/j.chemosphere.2016.03.070 27045633

[B31] Wang Z Z. Wang H. Wang H. Li Q. Li Y. (2019). Heavy Metal Pollution and Potential Health Risks of Commercially Available Chinese Herbal Medicines. Sci. Total Environ. 653, 748–757. 10.1016/j.scitotenv.2018.10.388 30759600

[B32] Xiao K. Song M. Liu J. Chen H. Li D. Wang K. (2018). Differences in the Bioaccumulation of Selenium by Two Earthworm Species (Pheretima Guillemi and *Eisenia fetida*). Chemosphere 202, 560–566. 10.1016/j.chemosphere.2018.03.094 29597172

[B33] Yan C. Yang F. Wang Z. Wang Q. Seitz F. Luo Z. (2017). Changes in Arsenate Bioaccumulation, Subcellular Distribution, Depuration, and Toxicity in *Artemia salina* Nauplii in the Presence of Titanium Dioxide Nanoparticles. Environ. Sci. Nano 4 (6), 1365–1376. 10.1039/c6en00621c

[B34] Zhou G.-W. Yang X.-R. Sun A.-Q. Li H. Lassen S. B. Zheng B.-X. (2019). Mobile Incubator for Iron(III) Reduction in the Gut of the Soil-Feeding Earthworm Pheretima Guillelmi and Interaction with Denitrification. Environ. Sci. Technol. 53, 4215–4223. 10.1021/acs.est.8b06187 30882209

[B35] Zhu F. Wang X. Fan W. Qu L. Qiao M. Yao S. (2013). Assessment of Potential Health Risk for Arsenic and Heavy Metals in Some Herbal Flowers and Their Infusions Consumed in China. Environ. Monit. Assess. 185, 3909–3916. 10.1007/s10661-012-2839-y 22983610

[B36] Zuo T. T. Li Y. L. He H. Z. Jin H. Y. Zhang L. Sun L. (2019). Refined Assessment of Heavy Metal-Associated Health Risk Due to the Consumption of Traditional Animal Medicines in Humans. Environ. Monit. Assess. 191 (3), 171. 10.1007/s10661-019-7270-1 30783770

[B37] Zuo T. T. Li Y. L. Jin H. Y. Gao F. Wang Q. Wang Y. D. (2018). HPLC-ICP-MS Speciation Analysis and Risk Assessment of Arsenic in Cordyceps Sinensis. Chin. Med. 13 (1), 19. 10.1186/s13020-018-0178-9 29686726 PMC5902960

